# Iron in the NEEM ice core relative to Asian loess records over the last glacial–interglacial cycle

**DOI:** 10.1093/nsr/nwaa144

**Published:** 2020-06-26

**Authors:** Cunde Xiao, Zhiheng Du, Mike J Handley, Paul A Mayewski, Junji Cao, Simon Schüpbach, Tong Zhang, Jean-Robert Petit, Chuanjin Li, Yeongcheol Han, Yuefang Li, Jiawen Ren

**Affiliations:** State Key Laboratory of Earth Surface Processes and Resource Ecology, Beijing Normal University, Beijing 100875, China; State Key Laboratory of Cryospheric Science, Northwest Institute of Eco-Environment and Resources, Chinese Academy of Sciences, Lanzhou 730000, China; Climate Change Institute, School of Earth and Climate Sciences, University of Maine, Orono, ME 04469, USA; Climate Change Institute, School of Earth and Climate Sciences, University of Maine, Orono, ME 04469, USA; Key Laboratory of Aerosol Science and Technology, SKLLQG, Institute of Earth Environment, Chinese Academy of Sciences, Xi’an 710061, China; Climate and Environmental Physics, Physics Institute, University of Bern, Bern 3012, Switzerland; Institute of Tibetan Plateau and Polar Meteorology, Chinese Academy of Meteorological Sciences, Beijing 100081, China; Institut des Geosciences de I’Environment (IGE), University Grenoble Alpes, Grenoble F38000, France; State Key Laboratory of Cryospheric Science, Northwest Institute of Eco-Environment and Resources, Chinese Academy of Sciences, Lanzhou 730000, China; Korea Polar Research Institute, Incheon 21990, Korea; State Key Laboratory of Cryospheric Science, Northwest Institute of Eco-Environment and Resources, Chinese Academy of Sciences, Lanzhou 730000, China; State Key Laboratory of Cryospheric Science, Northwest Institute of Eco-Environment and Resources, Chinese Academy of Sciences, Lanzhou 730000, China

**Keywords:** Greenland NEEM ice core, iron fertilization, carbon dioxide, Chinese loess, glacial–interglacial cycle

## Abstract

Mineral dust can indirectly affect the climate by supplying bioavailable iron (Fe) to the ocean. Here, we present the records of dissolved Fe (DFe) and total Fe (TDFe) in North Greenland Eemian Ice Drilling (NEEM) ice core over the past 110 kyr BP. The Fe records are significantly negatively correlated with the carbon-dioxide (CO_2_) concentrations during cold periods. The results suggest that the changes in Fe fluxes over the past 110 kyr BP in the NEEM ice core are consistent with those in Chinese loess records because the mineral-dust distribution is controlled by the East Asian deserts. Furthermore, the variations in the dust input on a global scale are most likely driven by changes in solar radiation during the last glacial–interglacial cycle in response to Earth's orbital cycles. In the last glacial–interglacial cycle, the DFe/TDFe ratios were higher during the warm periods (following the post-Industrial Revolution and during the Holocene and last interglacial period) than during the main cold period (i.e. the last glacial maximum (LGM)), indicating that the aeolian input of iron and the iron fertilization effect on the oceans have a non-linear relationship during different periods. Although the burning of biomass aerosols has released large amounts of DFe since the Industrial Revolution, no significant responses are observed in the DFe and TDFe variations during this period, indicating that severe anthropogenic contamination has no significant effect on the DFe (TDFe) release in the NEEM ice core.

## INTRODUCTION

Wind-borne mineral dusts impact Earth through biogeochemical supply, as they contain micronutrients (such as iron (Fe)) that fertilize the marine ecosystems [1–3]. In this study, we focus on iron, as it plays an important role in the oceanic biogeochemical cycle by limiting the primary productivity of phytoplankton and affects the carbon cycle by driving atmospheric CO_2_ drawdown [4,5]. East Asian deserts are considered the second greatest dust sources in the world, hence a large amount of dust emanates from these regions, particularly during boreal spring, and is deposited across the North Pacific Ocean (NPO). The dust often mixed with high concentrations of Fe and pollutants. For instance, a study of modern air-pollution aerosols over the East China Sea demonstrated that anthropogenic emissions (acidic sulfate) produce more bioavailable iron for ocean ecosystems [6]. Recently, models and observations compiled from field campaigns have identified that pyrogenic aerosols are the primary type of aerosols with high Fe solubility [7,8]. More importantly, the input of bioavailable Fe into the subarctic Pacific Ocean clearly enhances the phytoplankton growths and offers a direct link between East Asian mineral dust and NPO climate systems [9]. The dust froms East Asian deserts forms one of the largest and most persistent plumes globally and East Asia has been identified as the dominant source of dust transported to the Greenland ice sheet [10]. Therefore, Fe records from Greenland-ice-sheet ice cores can provide a wealth of modern and paleoclimatic information on human activity, land and the ocean, and can be used to integrate the spatio-temporal distribution with the transport dynamics of Asian dust.

Large amounts of data on dissolved Fe in seawater have been published by the international GEOTRACES program, enabling elucidation of the distributions of Fe in seawater at the global scale and greatly increasing our knowledge of the marine geochemistry of this element. However, available data are still limited for paleoclimate studies [11]. Current literature suggests that the ice sheets may release large amounts of dissolved Fe to the oceans via meltwater under future warming scenarios. Moreover, the anthropogenic activities may have resulted in a doubling or even tripling of atmospheric dissolved Fe transport to the oceans since the Industrial Revolution [12,13]. The Greenland GRIP, GISP2 and NGRIP ice cores from the Northern Hemisphere (NH) have provided centennial-scale records of δ^18^O and dust high-resolution data and supplied relatively well-constrained chronologies that serve as a master record for past climatic changes during the last glacial–interglacial cycle [14,15]. Previous studies have mostly focused on dissolved Fe records from the Antarctic ice cores [16–18]. Recent work suggests that Fe-related processes likely account for 5–30 ppmv of the change in the CO_2_ concentrations observed over glacial–interglacial transitions in the Antarctica ice core (i.e. 80–100 ppmv) [19]. So far, no high-resolution records of Fe data have been available from the NH ice cores for determining the relationships between the dust/Fe and CO_2_. In fact, Fe records from land, ocean and ice cores provide a wealth of paleoclimatic information for integrating the spatio-temporal distribution and the transport dynamics of aeolian dust in the NH. Thus, accurate evaluations of the past and present atmospheric deposition of Fe in the NH are needed.

A 2540-m-long ice core was drilled at the NEEM site, Greenland (77.45°N, 51.06°W, surface elevation 2450 m) during 2008–12 [20]. Detailed information on the NEEM ice core can be found in the Supplementary Fig. 1. The concentration of dissolved Fe (DFe, including the dissolved phase and colloidal fraction, <0.2 or 0.45 μm) in seawater has been widely used to gauge the biological availability of Fe to the algae [21]. Therefore, the operationally defined measurements of filtrates that pass through 0.45-μm filters mainly represent the dissolved phase and the colloidal fraction in this study. We first present the dissolved and total dissolved Fe data over the past 110 kyr BP in the NEEM ice core from northern Greenland. The positive correlations among the NEEM ice core, Asian loess records, oceanic productivity and atmospheric CO_2_ on different timescales strongly suggest that the dust and Fe have played important roles in the biogeochemical cycles in the NH over the past 110 kyr BP.

## RESULTS AND DISCUSSION

### Fe records in the NEEM ice core over the past 110 kyr BP

**Figure 1.**
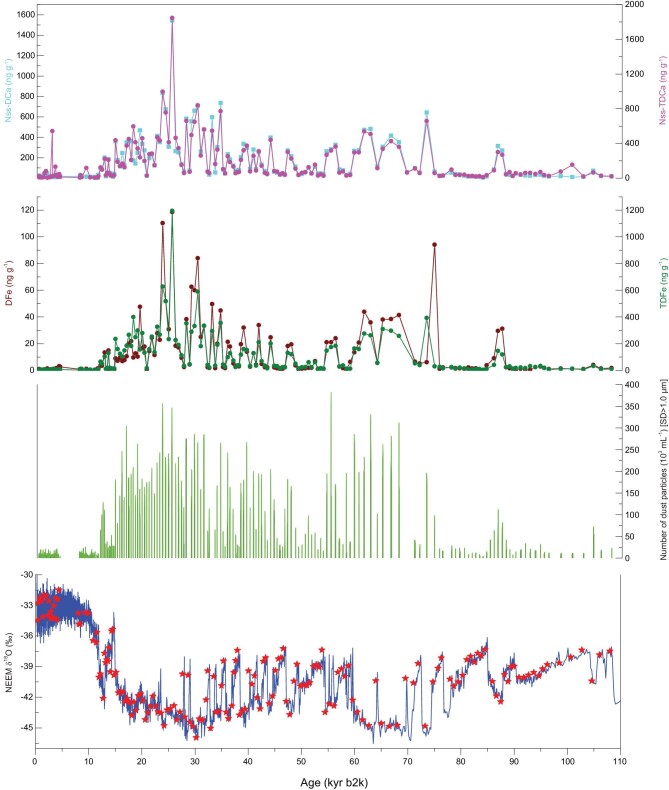
NEEM δ^18^O data from the NEEM Scientific Committee. Dust-particle, Nss-DCa and Nss-TDCa concentrations over the past 110 kyr BP. The red stars represent the data from ice samples used in this study.

To provide a better understanding of the Fe and dust records in the NEEM deep ice core over the past 110 kyr BP, Figure 1 presents a comparison of the dust number and non-sea salt (nss-)Ca concentrations in a discrete profile in the NEEM ice core (see the ‘Methods’ section). The number of dust particles exhibits significant fluctuations, but its pattern is extremely similar to the Ca and Fe variations. The DFe concentration varies from 0.01 to 118.53 ng g^−1^ (with an average of 12.05 ng g^−1^ and standard deviation of 19.58 ng g^−1^) and the TDFe concentration varies from 1.5 to 1194.5 ng g^−1^ (with an average of 101.38 ng g^−1^ and a standard deviation of 148.1 ng g^−1^) over the past 110 kyr BP. A regression line fitted to a plot of DFe versus TDFe has an *R*^2^ value of 0.80 (*P* = 0.01) over the past 110 kyr BP. Irrespective of the Fe (Ca) species, the cold climate conditions (such as those during the last glacial maximum (LGM)) are characterized by higher numbers of dust particles and Fe (Ca) concentrations, and warmer periods (such as the last interglacial period and the Holocene) were accompanied by lower Fe concentrations. The concentrations of dust particles and Ca were clearly and negatively correlated with the δ^18^O values in the NEEM ice core, which is consistent with the results from the earlier NGRIP, GRIP and GISP2 ice cores (Supplementary Fig. 2) [22–24]. The dust-particle and Fe concentrations show an extreme variation, reaching their maxima during the LGM. The nss-DCa concentrations are closely correlated with the nss-TDCa concentrations (*R*^2^ = 0.91, *P* = 0.01). Meanwhile, the nss-DCa and nss-TDCa concentrations are highly correlated with the synchronous variations between the DFe and TDFe concentrations in the NEEM over the past 110 kyr BP (*R*^2^ = 0.87 and 0.93, respectively, with *P* = 0.01). Thus, the DFe record exhibits the same characteristics as the aeolian deposition.

### Potential dust provenances for Greenland ice cores in comparison to Chinese loess records

Nss-K recorded in the GISP2 ice core during the Holocene is associated with the long-distance transport of the K-rich dusts from Central Asia to the Greenland ice sheet [25,26]. Sr–Nd isotopic data from the potential Chinese dust sources correspond well with the isotopic measurements of snow and ice samples at high-elevation interior sites on the Greenland ice sheet. A previous study argued that dust transported long distances from East Asian deserts represents the majority of the dust deposited on the Greenland ice sheet [27]. For instance, a comparison has been made between insoluble samples from Greenland ice cores and possible dust source areas, and the results suggest that East Asia was the main source area [28,29]. Although a central European source cannot be excluded as another potential source in a recent study, East Asian deserts appear to be the most likely dominant source of Greenland dust over the last glacial period, supported by new clay mineral and Sr–Nd isotope data [30]. Furthermore, Asian dust aerosols have been shown to be able to be transported from north-western China to the Arctic within 5 days by simulation [31]. However, these records and studies used discrete samples and short timescales. The loess deposits of the Chinese Loess Plateau (CLP) shed light on the links between dust sources and records in the Greenland ice sheet, thus providing important information for understanding long-distance dust transport in the NH [32].

The variation in the mean grain size (MGS) gradually decreased from the Jingyuan (–5.9 to 5 μm, 36°21^′^N, 104°37^′^E) to the Xifeng (–2.9 to 3.8 μm, 35°47^′^N, 107°36^′^E) and Weinan (–1.4 to 2.3 μm, 34°21^′^N, 109°31^′^E) loess sections, suggesting that the strength of the westerlies weakens in this direction from west to east [33]. This spatial pattern is consistent with the observed south-eastward decrease in orbital-scale MGS variations, implying that the amplitude of the rapid MGS decreases can be considered a direct indicator of the intensity of the westerly circulation. Meanwhile, the intensity of the westerly circulation provides the driving force necessary to transport mineral dust from Asia to high-latitude regions of the NH. The magnitude of fluctuations associated with the 20 Dansgaard–Oeschger (D-O) and 6 Heinrich (H) events recorded in the three Chinese loess profiles decreases from west to east during the last glacial period [33]. Previous studies have indicated that the NGRIP dust concentrations can be used as a qualitative proxy for evaluating dust-storm activities in East Asia [32,34]. In addition, the Xifeng aeolian dust record resembles the δ^18^O record of core V21–146 (37°41^′^N, 163°02^′^E) from the north-western Pacific [35]. These results imply that aeolian dust is transported by westerlies from the Central Asian deserts and deposited in the NPO and even in Greenland. Thus, similar climate events have occurred on various spatio-temporal scales in the NH [35–37].

**Figure 2.**
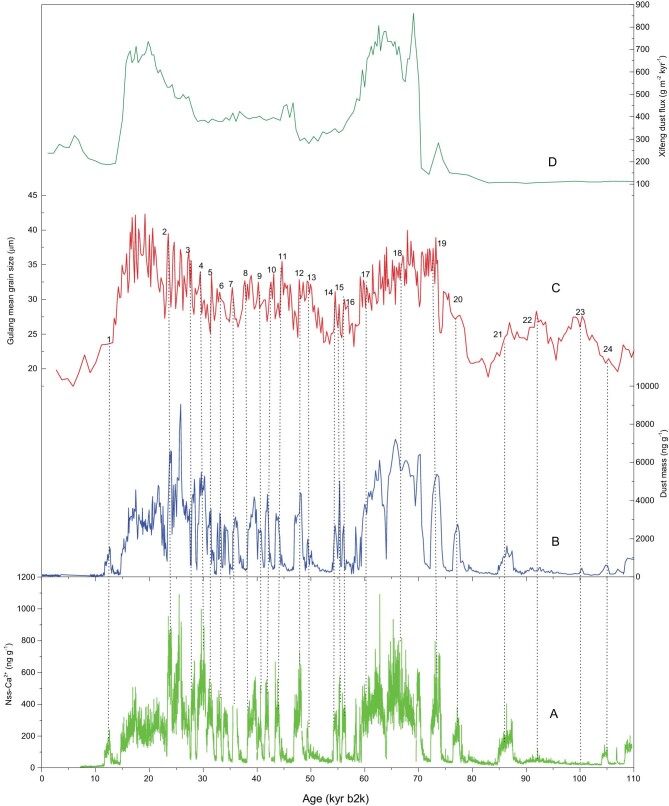
Comparison of the concentrations of Nss-Ca^2+^ (A) [41], the dust mass (B) in the NEEM ice core from this study, the median grain size in the Gulang record (C) [39] and the Xifeng dust fluxes (D) [38]. The dotted lines show the correlation of 24 D-O cycles between the NEEM ice core and the Gulang loess.

Figure 2A and B presents the comparison between the nss-Ca^2+^ and dust-mass concentrations (which mainly reflect atmospheric dust loading) in the NEEM ice core with the dust fluxes, obtained from the Xifeng loess section (resolution: 290–2410 yr) and the dust MGS (resolution: 150–1050 yr) in the Gulang loess section (37.49°N, 102.88°E, 2400 m a.s.l.) from the CLP (Supplementary Fig. 1) [38,39]. Evidence from Xifeng suggested a broad coupling between Asian and Antarctic climates at the glacial–interglacial scale [38,40]. Although the dust resolution in the Xifeng loess section is relatively low, there is a broad similar trend between nss-Ca^2+^ concentrations in NEEM ice core and dust fluxes. The Gulang loess section is located in the north-western part of the CLP, on the margin of the Tengger desert, characterized by a high sedimentation rate and weak pedogenesis, and is hence very sensitive to climate change on orbital and millennial scales [39]. Compared with previous data from the Greenland ice cores, the high-resolution (1-mm) nss-Ca^2+^ data, obtained from the NEEM ice core through continuous-flow analysis, enable the recording of centennial- to millennial-scale events [41]. On glacial–interglacial timescales, the results from these four ice cores further demonstrate synchronous climatic events (Supplementary Fig. 2). Higher nss-Ca^2+^ and dust-mass concentrations in the NEEM ice core correspond to the increased values of MGS in the Gulang loess section at 24 D-O events (Fig. 2A–C). Thus, a strong correlation can be established between the climatic conditions in Greenland and Asia over the past 110 kyr BP on a centennial timescale. Specifically, the two periods of high dust concentrations during the last glacial period, at 70–58 and 35–15 kyr, are evident, both in the NEEM ice core and in the Gulang and Xifeng loess sections.

### Fe records in NEEM and potential links to CO_2_ concentrations

**Figure 3.**
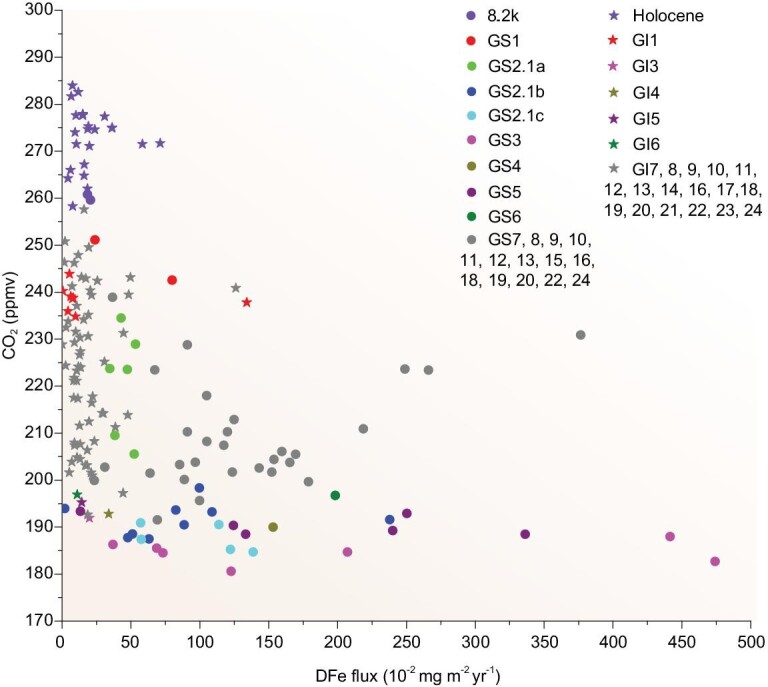
Scatter plot of DFe flux versus the CO_2_-concentration record from Antarctic ice cores. The CO_2_ data presented are from Antarctica ice cores, which can be found at https://www.ncdc.noaa.gov/data-access/paleoclimatology-data.

**Figure 4.**
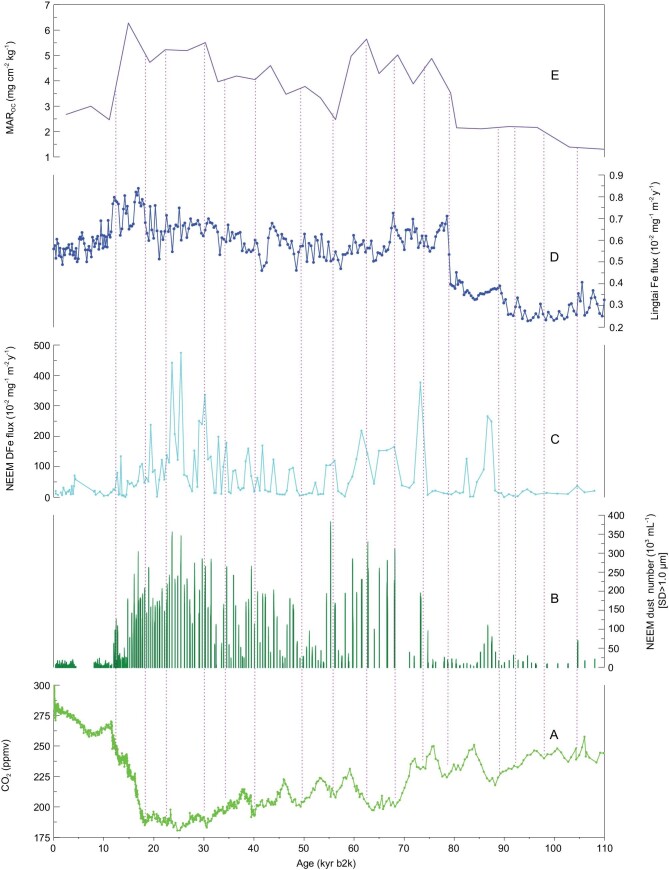
DFe fluxes and dust-particle concentrations recorded in the NEEM ice core, Fe fluxes from the Lingtai and Xifeng loess profiles, paleoproductivity represented by the variations of MAR_OC_ in the H3571 core from the North Pacific Ocean and atmospheric CO_2_ recorded in Antarctica ice cores over 110 kyr BP. (A) CO_2_ concentration in the Antarctic ice cores from https://www.ncdc.noaa.gov/data-access/paleoclimatology-data; (B) the NEEM dust-particle concentration (this study); (C) the NEEM DFe flux (this study); (D) Fe flux in the Lingtai loess [42]; (E) H3571 MAR_OC_ [44]. Magenta dotted lines mark low CO_2_ concentrations, which coincide with high Fe fluxes.

The Greenland Stadials (GSs) and Greenland Interstadials (GIs) are expressions of D-O events and represent cold and warm climatic phases, respectively, in the North Atlantic region [14]. An evident relationship is identified between the DFe fluxes in the NEEM ice core and the new high-resolution CO_2_ data from the Antarctic ice cores (ftp://ftp.ncdc.noaa.gov/pub/data/paleo/icecore/). The higher CO_2_ levels of depositions during the Holocene are clearly associated with the lower DFe fluxes (Fig. 3); specifically, the CO_2_ concentrations oscillate above 260 ppmv, whereas the Fe fluxes have a mean Holocene value of 20.37 × 10^−2 ^mg m^−2^ yr^−1^. As shown in Fig. 3, this pattern most likely indicates that the CO_2_ decreases simultaneously with peaks in Fe fluxes during the GSs. Indeed, the GS2.1 (a, b and c), GS3, GS4 and GS5 exhibit lower CO_2_ levels and the centennial-scale CO_2_ oscillations coincident with the changes in the DFe fluxes may be recorded in the NEEM ice core. In contrast, the low Fe fluxes evidently fail to respond significantly to the low CO_2_ concentrations during the GIs. Thus, the Fe fertilization during warm periods cannot explain the atmospheric CO_2_ variations. In the Holocene, the Fe-driven changes in atmospheric CO_2_ terminated with rapid increases in CO_2_ concentration (from 245 to ∼285 ppmv), whereas the Fe fluxes remained relatively constant (Fig. 4A). Therefore, although it is difficult to quantify the CO_2_ changes based on the NEEM ice core, due to the different resolutions of the CO_2_ records and Fe concentrations, the NEEM ice-core record suggests that the Fe fertilization effect has produced more significant changes during the GSs events than during the GIs events (Fig. 3). Thus, Fe fertilization cannot explain the atmospheric CO_2_ variations during warm periods.

The Asian dusts account for approximately two-thirds of the biologically available Fe in high-nutrient low-chlorophyll regions in the NPO [42]. The dusts from the Asian deserts were transported in the upper-level westerly jet and resulted in the deposition of Fe in the NPO and even in the Greenland ice sheet. Therefore, the DFe in the NEEM ice core may reflect the bioavailable Fe released from mineral dust in the NH. A previous study has shown that decreases in the inputs of dust from Asian deserts to the northern Pacific can change the atmospheric CO_2_ concentrations by ≤8 ppm [43]. However, so far, no studies of deep ice cores in Greenland have considered the effects of aeolian dust Fe on CO_2_ concentrations. Based on the Fe fluxes estimation from the Lingtai-loess record from the CLP (subsamples were taken at intervals of 4 and 2 cm, which correspond to a temporal interval of 184–570 yr), a direct paleoclimatic link has been established between the aeolian dust (Fe) and CO_2_ in Fig. 4A–C. The Lingtai-loess sections (35°04^′^N, 107°39^′^E) are located on the western side of the CLP and include the classic loess profiles (Supplementary Fig. 1) [38,42]. As presented in Fig. 4D, the Fe flux varies from 0.23 × 10^−2^ to 0.84 × 10^−2 ^mg m^−2^ yr^−1^ in the Lingtai-loess section, with the higher rate of the Fe fluxes corresponding to glacial stages and lower Fe fluxes corresponding to interglacial periods [42]. The Fe fluxes in the NEEM ice core and Lingtai-loess section decreased to a minimum during the last interglacial period and increased during the last glacial period. The Fe fluxes decreased markedly during the Holocene, but that flux was still higher than that during the last interglacial period in the Lingtai loess [42]. The DFe flux in the NEEM ice core during the Holocene is similar to that during the last interglacial period. These observations provide quantitative constraints on the input of aeolian iron from Asia to the ocean during cold periods. Therefore, a comparison of the NEEM Fe data with the Lingtai loess records reveals a good correlation between the rapid changes in the Fe concentration. These climatic events can be identified in the NEEM ice core, corresponding to the Holocene–Younger Dryas transition (11.5 kyr) and the (Marine Isotope Stage) MIS 4/5 boundary (75 kyr). Additionally, significant low-CO_2_ periods are observed in Fig. 4A. These results constrain the quantity of aeolian Fe transported from Asian deserts to the Greenland ice sheet.

The marine sediment core H3571 (34°54^′^N, 179°42^′^E) was collected from a mid-latitude location in the NPO. The mass accumulation rate (MAR) of organic carbon (MAR_OC_) in this core is used as a proxy for marine paleoproductivity in the NPO, with a low temporal resolution of 1670–6570 yr [44]. Good correlations are observed between the Al and quartz concentrations in aerosols, and MAR indicates that the aluminosilicate minerals in the sediments are transported from the Asian continent mainly by wind. During the past 110 kyr BP, MAR_OC_ exhibits two prominent maxima in oxygen isotope stages (OISs) 2 and 4, as well as relatively high values in the middle and late parts of OIS 6, corresponding to the peaks in the Fe fluxes in both the NEEM ice core and the Lingtai-loess section. This inverse relationship between the atmospheric CO_2_ and the productivity implies that the biological pump operating in the NPO may have affected the atmospheric CO_2_ levels over the past 110 kyr BP. Thus, the results from the NEEM ice core can provide a more representative estimate of DFe fluxes in the NPO during the past 110 kyr BP. Furthermore, the oceanic biological pump that transports carbon from the surface to the deep sea has been identified as a key modulator of atmospheric pCO_2_ over glacial–interglacial timescales [45]. The DFe fluxes in the NEEM ice core can be compared to the measurements of atmospheric CO_2_ (from Antarctic ice cores), Fe (from CLP loess records) and the MAR_OC_ ocean productivity proxy (from marine sediments of the NPO). This result supports the hypothesis that changes in the atmospheric CO_2_ are partly due to the fluctuations of iron deposition, based on data from Asia, the Pacific and Greenland. Thus, high oceanic productivity should have occurred during these glacial periods when the DFe concentrations were high, whereas low productivity should have occurred during the Holocene and other interglacial periods, when the DFe concentrations were lower (Fig. 4C). The records show a similar pattern of variation over the past 110 kyr BP and provide further evidence for a link between the dust particles and the Fe in the NH (Fig. 4B and C). The differences in both mediums also suggest that bioavailable Fe played a role in modulating atmospheric CO_2_ during cold periods. Unfortunately, because of the low resolutions in this study, the detailed climatic events cannot be further discussed.

Millennial-scale climate variations during the Pleistocene are manifested differently in various climate archives. Examples include the cold Heinrich (H) events recorded in the North Atlantic sediments and the D-O events recorded in Greenland ice, Chinese stalagmites and loess sections [37,45–47]. Notably, the δ^18^O records from the stalagmites from the caves Hulu (32°30^′^N, 119°10^′^E) and Sanbao (31°40^′^N, 110°26^′^E) (in eastern and central China, respectively) resemble the δ^18^O record of the NEEM ice core [47,48] (Supplementary Fig. 3). A comparison of millennial-scale events in the δ^18^O records in stalagmites from Hulu and Sanbao caves reveals general similarities with the NEEM ice core over the past 110 kyr BP (i.e. 24 abrupt D-O warming events; see Supplementary Fig. 3 for more details). The two records are synchronous with summer (July) insolation at 65°N [49,50], which supports the idea that Fe records in Central Asian loess and the Greenland ice core responded predominantly to changes in NH summer insolation at orbital timescales [47]. These couplings at various scales demonstrate the links between Central Asia and Greenland over 110 kyr BP.

### Fe variability between LGM and Holocene

**Figure 5.**
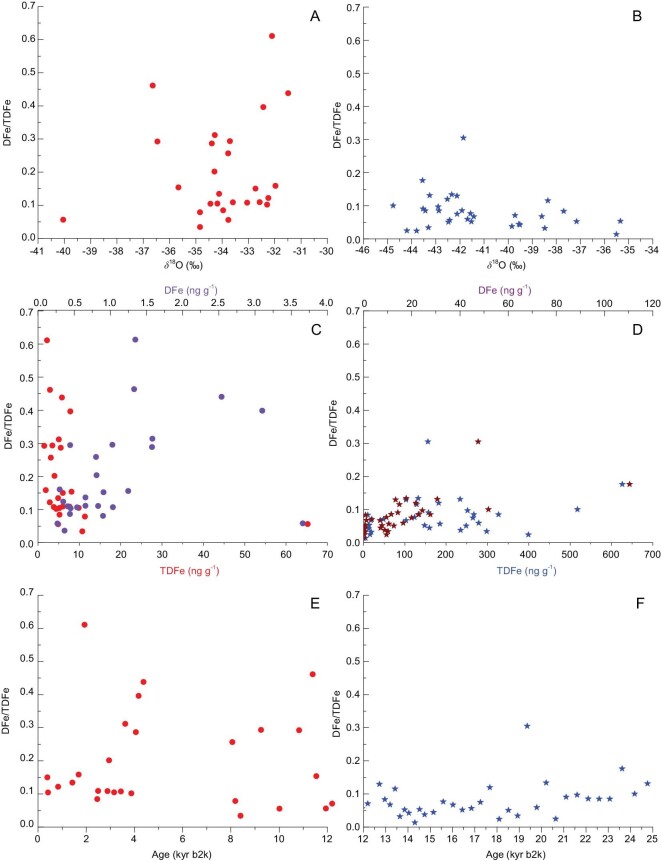
Variability of DFe/TDFe with δ^18^O, DFe and TDFe during 393–12 000 (A, C and E) and 12 000–24 765 yr b2k (B, D and F), respectively, in the NEEM ice core. Ice from an identical age has been subsampled to investigate the variability in the DFe/TDFe ratios.

To assess the differences between the LGM and the Holocene, the DFe, TDFe and δ^18^O values and the DFe/TDFe ratios in the NEEM ice core are correlated in Fig. 5. The DFe/TDFe ratios are lower during the LGM compared to that during the Holocene. The DFe/TDFe ratios exhibit an inverse hyperbolic relationship with the TDFe concentrations, whereas the ratios show a slight increase, as the DFe concentrations increase in the samples from the Holocene period (Fig. 5C). A significant feature is the general trend of the DFe/TDFe ratio over time, i.e. the high values (>19%), restricted to times when δ^18^O >–37‰ and TDFe <5 ng/g during the Holocene (Fig. 5A). Moreover, a general trend of low DFe/TDFe ratios (<0.15) is observed from 25 to 12 kyr before AD 2000 (b2k) (Fig. 5A). High DFe/TDFe ratios occur only after 12 kyr b2k (the Holocene), except in one sample that was dated to 19.36 kyr b2k. During the LGM, the DFe/TDFe ratios remain stable with the DFe (TDFe) concentration (Fig. 5D). The mean TDFe fluxes decrease from 12.46 mg TDFe m^−2^ yr^−1^ during the LGM (25–18 kyr b2k) to 1.2 mg TDFe m^−2^ yr^−1^ at the onset of the Holocene (10.835–0.393 kyr b2k) (Fig. 5E and F). The TDFe fluxes (6.29–47.78 mg m^−2^ yr^−1^; mean 17.58 mg m^−2^ yr^−1^) are up to 20 times higher than the total digested Fe fluxes (0.31–2.02 mg m^−2^ yr^−1^; mean 0.77 mg m^−2^ yr^−1^) previously observed in the Antarctic EPICA Dome C (EDC) ice core spanning the LGM (26–21 kyr b2k) [51], which is attributed to the larger dust areas in the NH.

**Figure 6.**
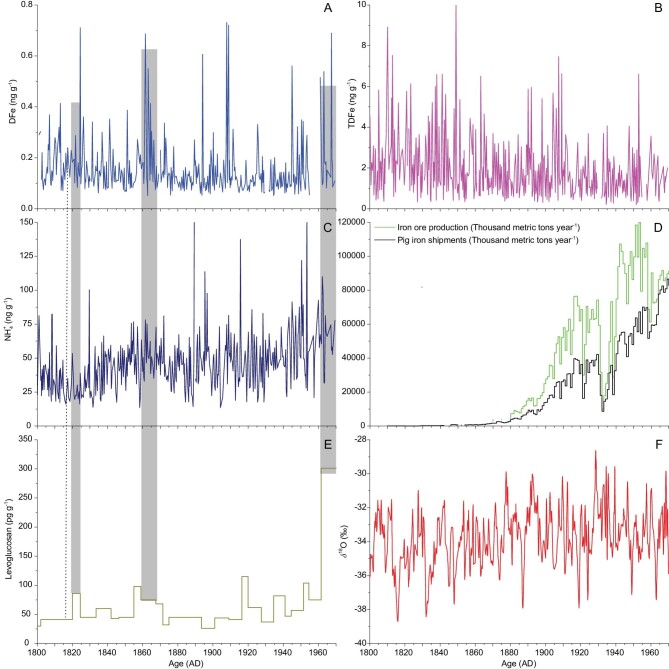
DFe, TDFe and NH_4_^+^ concentrations (A–C) and δ^18^O values in the NEEM 2009 shallow Core 1 (F), the levoglucosan concentration in NEEM 2011 S1 [62] (E) and the iron-ore production and pig-iron shipments data (D) from the USA between 1800 and 1970 [66]. The dashed line in parts (A, C and E) represents the Tambora eruption in 1815. Gray shadings represent the peaks between DFe and NH_4_^+^ and levoglucosan concentrations.

As shown in Fig. 6A and B, the range of the fluctuation margin in the DFe/TDFe ratios in the NEEM ice core during 1870–1969 is 1.7%–64%, with an average of 12%, and there is an increasing trend during 1945–1967 (2.4%–64%, mean 17%). The DFe/TDFe values fluctuate between 3.4% and 61% in the Holocene samples (393–11 549 yr BP), with an average of 19%. Moreover, the moderate maxima are observed in the interglacial regions, with values that are approximately twice those observed in the LGM (18 099–24 765 yr BP) samples (2%–30%, with an average of 10%) (Supplementary Fig. 4). The high variable Fe solubility (1%–42%) is also observed in the Dome C ice core during the LGM [51]. However, modeling studies assume a constant solubility of 2% in models [52]. Although the higher Fe solubility of the glacial dust has been examined in the LGM simulation in a previous study [53], it should be noted that the Fe solubility is different in warm periods (Holocene and interglacial periods). Multiple factors control Fe solubility. Physical processes can explain the variations in the solubility of Fe in aerosols because larger particles are removed during long-range transport, altering the mineral aerosol size distribution [54]. For the NEEM deep ice-core samples, a significantly positive correlation (*R*^2^ = 0.63, *N* = 155) is observed between the DFe and mass of fine particles (0.8–2 μm), while a weak positive correlation (*R*^2^ = 0.42, *N* = 155) is identified between the DFe and mass of coarse particles (2.2–5 μm). The mass ratios between the fine and coarse particles are presented in Supplementary Fig. 4. Although no significant correlation is observed, the patterns between the DFe (TDFe) ratios and the fine/coarse ratios are similar. Thus, the DFe concentration can release as the dust-transport distance increases, hence resulting in the abundance of small-particle depositions that are relatively rich in soluble iron [55–57]. When the lower dust concentrations are present, although the modal size of the mineral dust is smaller, the corresponding surface-area-to-volume ratio is higher; thus, a greater proportion of the Fe is present near the surfaces of the particles and can be dissolved [56]. Consequently, variations in the dust-grain size likely result in variations in the DFe (TDFe) concentrations.

As observed on various temporal scales, the dust-particle size is smaller during the warm Holocene and interglacial periods than during the last glacial period. For example, the modal sizes of the dust in the LGM and Holocene sections of the GRIP ice core are 1.94–2.02 and 1.60–1.80 μm, respectively [24]. The size (μ) distribution has a mode of 2.5 μm in the NEEM ice core during the LGM, with generally coarser particles ≤10 μm. While there is a mode of 2.7 μm in the NEEM ice core during the Holocene, the coarser particle is <6 μm. To sum up, based on our findings, the DFe/TDFe ratios in the NEEM ice core are higher during the Holocene compared to the LGM, due to the physical process involved in the long-distance transportation of mineral dust (aerosols). Biomass burning and aerosols associated with modern air pollution may have contributed to the observed changes in the Fe solubility [7,55,58]. The inverse hyperbolic relationship between the DFe/TDFe ratios and the TDFe during the Holocene may be attributed to the mixing of low-Fe-solubility mineral dust with other soluble Fe aerosols from various sources and processes [59]. Therefore, these results indicate that the effects of Fe fertilization become more complex during the warm periods, suggesting that there are other factors acting as amplifiers and significantly increasing the DFe/TDFe ratio during the Holocene.

### Fe and biomass-burning proxies in the NEEM shallow ice core

The DFe (TDFe) and other proxies were measured in the NEEM 2009 shallow Core 1 (Core 1) in order to focus on the relationship between anthropogenic pollutants and DFe (TDFe) concentrations (Fig. 6). The cold periods (1810s and 1830s) correspond to high TDFe concentrations. Although it is not statistically insignificant, there are decreasing TDFe variations during 1800–1969. The minimum δ^18^O values in Core 1 occur in 1816, as a result of the Tambora eruption in 1815; however, no remarkable increase is observed in the concentrations of DFe and TDFe (Fig. 6A, B and F). The DFe and TDFe concentrations range from 0.05 to 0.73 ng g^−1^ (mean value 0.16 ng g^−1^) and from 0.2 to 10.2 ng g^−1^ (mean value 1.9 ng g^−1^), respectively, in Core 1. A weak positive relationship (*R*^2^ = 0.25, *N* = 362) is observed between the DFe and TDFe concentrations, as well as a positive correlation (*R*^2^ = 0.50, *N* = 373) between the TDFe and TDCa concentrations, indicating that aeolian dust might have been the primary TDFe source during 1800–1969. However, the obvious positive relationship between DFe and total Fe is observed in the North Pacific and North Atlantic aerosol samples [8]. This difference may be attributed to DFe deposition fluxes and chemical components of the ice samples. First, the ice samples as liquid contain a lower amount of mineral dust than aerosol samples; therefore, the DFe and TDFe concentrations are lower in ice samples. Second, the pyrogenic aerosol samples contain many more anthropogenic pollutants, with a large amount of acidic pollutants such as sulfate, nitrate and oxygenated organic species. Furthermore, different degrees of atmospheric processing are involved in the Fe-containing aerosols [8]. Therefore, in comparison with the Fe record during 1800–1969 in Core 1, these results show that there are other DFe sources that need to be investigated.

Ammonium (NH_4_^+^) concentrations in the Greenland ice cores not only originated from North American (NA) soil emissions, but also combined with other pyrogenic aerosol tracers from NA wildfire events, which are often successfully associated with the biomass-burning events [60–62]. Many high fire years are identifiable by enhanced NH_4_^+^ concentrations in continental environmental archives [63]. Levoglucosan is also recently widely used as a biomass-burning marker in Greenland snows [64]. Modern aerosol evaluations suggest that the solubility of iron is enhanced when mixed with aerosols produced by biomass burning and acidic materials [6,55,58]. These biomass-burning markers link the biomass-burning aerosols with high Fe solubility in aerosols because DFe is released from pyrogenic Fe oxides [7]. To evaluate the impact of biomass burning on Fe solubility, data on the specific biomarker NH_4_^+^ and DFe concentrations are compared in NEEM and NGRIP ice cores (Supplementary Fig. 4). The terrestrial biogenic aerosol proxy of NH_4_^+^ between the NEEM and NGRIP ice cores is very similar. The NGRIP ice core indicated NH_4_^+^ background source concentrations from NA wildfire emission. The fire peak frequency showed a clear and immediate response to most DO events during MIS 3, with an approximate tripling of the NH_4_^+^ peak frequency [65]. However, DFe concentrations did not significantly increase the trend during MIS 3, indicating that the wildfire activity from NA does not make a possible contribution at this point.

Levoglucosan concentrations in the NEEM 2011 S1 ice core over the past 2000 years coincide with the biomass-burning aerosols from NA [62]. However, the DFe concentrations have no significant increasing trend (Fig. 6A). There is no correlation between the DFe and levoglucosan (NH_4_^+^) concentrations. In particular, there was a significantly increasing trend in the levoglucosan concentration since the 1960s in NEEM 2011 S1, which also corresponds to the DFe peak. In addition, there are three levoglucosan peaks (1820, 1865 and 1965) corresponding to three DFe peaks (Fig. 6A and E). Therefore, air-pollution aerosols from the biomass burning may have made a significant contribution during particular fire events. However, it may not be the main factor that has controlled DFe-release variations since the start of the industrial era. To further test the anthropogenic emissions effect on Fe release, Fig. 6D also presents the amount of iron-ore production and pig-iron shipments in the USA from 1800 to 1970 [66]. There is an abruptly increasing trend for them at the end of the nineteenth century and both decrease due to the great depression of the 1930s. Then, iron-ore production reached a maximum value in the 1950s, while pig-iron shipments have an upward trend since the 1930s. However, these patterns have absolutely different DFe variations. Therefore, the remarkable increase in anthropogenic pollutants since the industrial era has so far not been observed in the DFe (TDFe) NEEM ice record.

## CONCLUSION

The Fe fluxes in the NEEM ice core are evaluated in this study. Our results suggest that the Fe fluxes in the NEEM ice core are consistently greater than the Fe fluxes observed in the Antarctic ice core. Comparison between the iron records from the NEEM ice core and the Lingtai loess from the central CLP over the past 110 kyr BP indicates that the increasing rates of DFe deposition possibly contribute to the decreased levels of atmospheric CO_2_ during the GS intervals. This result is consistent with previous studies of the Fe records in the loess deposits and provides new insights for evaluating hypotheses related to Fe deposition during the last glacial–interglacial cycle in the NH. The DFe/TDFe ratios in the NEEM ice core present higher values during the Holocene and Industrial Revolution periods compared with those in the cold periods (i.e. the LGM), indicating that the Fe fertilization effect is more complex during the Holocene, due to the presence of different compositions of dust, with various grain sizes and other factors. The results also emphasize that the changes in the biological pump effect cannot be explained by a simple linear relationship with the glacial–interglacial changes in the atmospheric CO_2_. In particular, anthropogenic pollutants (including biomass burning) have a weak effect on DFe and TDFe variations, although they have increased markedly since the industrial era.

## Supplementary Material

nwaa144_Supplemental_File
