# Supplementary material for: Iron in the NEEM ice core relative to Asian loess records over the last glacial–interglacial cycle
Source: Natl Sci Rev. 2020 Jun 26;8(7):nwaa144. doi: 10.1093/nsr/nwaa144 (PMC8310736; doi:10.1093/nsr/nwaa144)
Supplement: nwaa144_Supplemental_File [file nwaa144_supplemental_file.doc]

**Supplementary Information**

**Iron in the NEEM ice core relative to Asian loess records over the last glacial-interglacial cycle**

Cunde Xiao^1,┼,*^, Zhiheng Du^2,┼,*^, Mike J Handley^3^, Paul A Mayewski^3^, Junji Cao^4^, Simon Schüpbach^5^, Tong Zhang^6^, Jean-Robert Petit^7^, Chuanjin Li^2^, Yeongcheol Han^8^, Yuefang Li^2^, Jiawen Ren^2^

**Methods**

The 2,538-m-deep NEEM ice core was drilled in north-western Greenland in 2009-2011, in conjunction with an international consortium of scientists from 14 nations, led by the University of Copenhagen, Denmark (http://neem.dk). The main goal of the project was to obtain climate records, spanning the last interglacial period, the Eemian, which occurred 115,000-130,000 years ago [1]. After processing the ice core sections, 166 discrete 3.5 cm×3.5 cm×20 cm section samples were collected in a shipping container, converted into a particle-free laboratory. Each section was opened in a class-100 HEPA clean bench, located in the main freezer at the Climate Change Institute of the University of Maine (CCI). Dating of the NEEM deep ice core samples was conducted according to the Greenland Ice Core Chronology 2005 (GICC05modelext-NEEM-1). The NEEM 2009 S1 shallow core was drilled at the NEEM site in northwestern Greenland. The total length of the ice core was 90.15 m. A total of 1862 samples (with resolutions of about 2.5 cm) were analyzed to determine their stable isotopic compositions, including oxygen (δ^18^O) and hydrogen isotopic compositions (δD), using Picarro L1102-i, L2130-i and L2140-i wavelength-scanned cavity ring-down spectrometers (Picarro Inc., USA) at the Korea Polar Research Institute. The NEEM 2009 S1 shallow core was melted using a continuous flow analysis (CFA) system for trace element analysis in Korea Polar Research Institute. In total, 778 samples (with resolutions of about 10 cm, including dissolved and total dissolved) were also measured with high-resolution inductively coupled plasma mass spectrometry (ICP-SFMS) at CCI in this study.

Among these samples of deep ice core, 24 samples (20-cm section) were collected from the Holocene, and 142 samples (5 cm cut from 20 cm section) were obtained from other periods. The ice samples were processed, as follows: First, the outermost 2-3 mm of each core section, which was heavily contaminated during drilling, was mechanically removed using a ceramic blade, cleaned with ultrapure deionized water. Then, the samples were kept frozen until they were thawed at room temperature in a clean laboratory. Subsequently, each sample was extracted using plastic pliers and carefully washed with deionized water to obtain clean samples. Only the innermost portions of the 166 samples (approximately 15 ml) were used for the ICP-SFMS analysis. For the ICP-SFMS analysis, first the bottles (LDPE, Fisher) were carefully cleaned by soaking them in 10% Optima grade HNO_3_ (Fisher) for one week, followed by rinsing the bottles three times with deionized water, soaking them for one week in deionized water, rinsing the bottles three times with deionized water, and drying under a class-100 dryer. The concentrations of contaminants in the blanks during the 24-hour procedure of acid leaching were low (including DI water, with average Fe content of 36 pg ng^-1^; DI water used to rinse the artificial ice samples, with average Fe content of 6 pg ng^-1^; and acid-cleaned sample bottles, with average Fe content of 25 pg ng^-1^).

To measure the concentrations of dissolved elements, cleaned and melted samples were filtered through polypropylene 0.45-μm filters (Whatman), and the concentrations of total dissolved elements were determined by acid-leaching the samples with HNO_3_ at the Climate Change Institute. Each sample was acidified to 1% using Optima double-distilled HNO_3_ in a class-100 HEPA clean bench, capped, shaken, and allowed to digest for six weeks after pouring into a set of acid-cleaned polypropylene vials. The Fe concentrations were determined using ICP-SFMS coupled with a Cetac Model ASX-260 autosampler. Interferences were minimized using an ESI Apex desolvating sample introduction system and a PFA-ST nebulizer. The trace element procedure in the ice core is described in detail in a previous study [2]. The analyses of acidified Antarctic snowmelt waters over a period of four to five months demonstrated that the Fe concentration tended to stabilize one month following acidification to pH 1.9 with hydrochloric acid [3].

To assess the roles of methodological differences on the measured trace element concentrations in ice cores at the Climate Change Institute, leached Antarctic snow samples were collected for 3 months at room temperature using nitric acid at concentrations of 0.1, 1.0 and 10.0% (v/v) [2]. At selected intervals (20 min, 24 hours, 5 days, 14 days, 28 days, 56 days, and 91 days), trace elements (including Fe) were measured using ICP-SFMS. The results showed that the slope of the 1% acid Fe concentration curve reached a steady state after one month. Although the dust particles were probably not completely dissolved during the acid-leaching period of 1–3 months, the digestion method can significantly contribute to contamination. For example, the procedural blanks for the digest method contained 1.9 ng g^-1^ Fe, which was two orders of magnitude greater than the Fe concentrations in the decontamination blanks (47 pg g^-1^ for Fe) [4]. The total Fe concentration was approximately 1 ng g^-1^ for the EDC ice core, even when the digestion method was used [5]. Therefore, if the ice core samples cover the glacial-interglacial cycle, a better method is to subject the samples to acid leaching for 1–3 months under the present instrumental conditions. In this study, all samples were acidified for at least six weeks (approximately 2 months). Dissolved and total dissolvable Fe and the other trace elements were analyzed by ICP-SFMS.

The dust concentrations in the NEEM deep ice core were measured by the University of Bern with continuous flow analysis with 1 mm resolution and at Univ. Grenoble Alpes, for concentration and size distributions, on 1.10 m (2 bags) average samples with a Coulter Mutisizer. The dust concentration data corresponded to the 20 cm values for Holocene samples and the 5 cm values for glacial samples in this study. The concentrations of nss-Ca^2+^ (nss-Ca^2+^=Ca^2+^-Rm•ss-Na^+^) were calculated using equation [4], where R_t_=1.78 and R_m_=0.038 are the average ratios of Ca^2+^/Na^+^ in the crust and marine systems, respectively. The NEEM deep ice core accumulation rate (20-yr resolution) was calculated and can be used to obtain DFe (TDFe) fluxes [6]. When it was assumed that 1% HNO_3_ (pH≈0.8) acid-leaching data are equivalent to data obtained from the pH 1 leaching method (based on ref. [2]), comparison of the 20 min and 1-month acidification data (HNO_3_, 1% (v/v)) showed that the Fe concentration increased from 0.402 ± 0.040 to 1.45 ± 0.187 ng ng^-1^ (an increase of 260%, ref. [2]). To compare the different TDFe data, a coefficient of 3 was multiplied by the previous TDFe data from the Antarctica ice cores. The accumulation rate in the deep parts of the NEEM ice core was calculated, and the record has a resolution of 20 yr. The accumulation rate can be used to calculate the fluxes of Fe and the other elements. Although the DFe/TDFe ratios in the NEEM ice core represent fractional Fe solubility, our calculated Fe solubility values may be overestimated because none of the samples were digested. However, by using a simple pH 1 acidification (at least 24 h), only approximately 30–65% of the total iron content in the Dome C ice core was observed [5]. It is noteworthy that DFe in the NEEM ice core was acidified for six weeks. Therefore, it could be generally compared to the previous results.


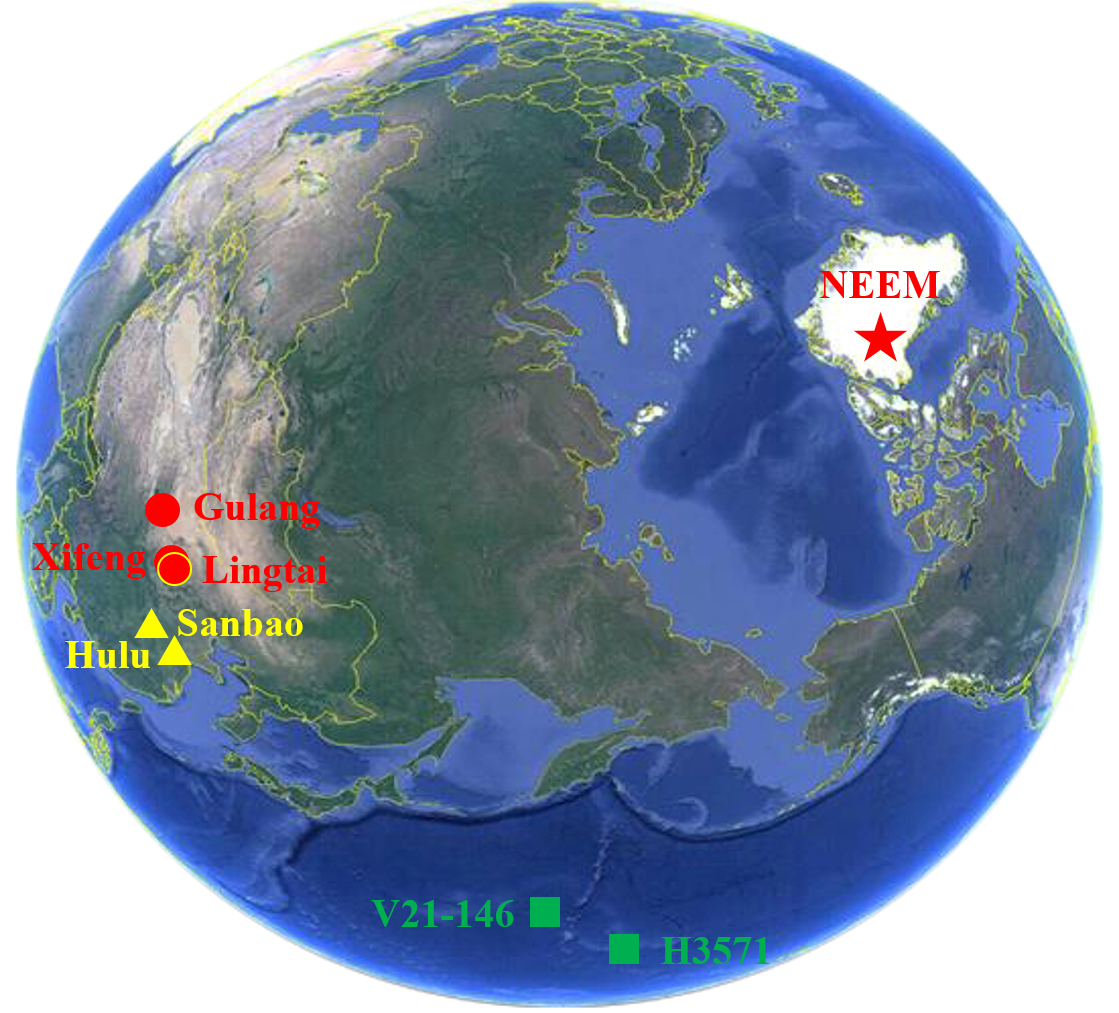


**Supplementary Figure 1. Locations of the different sampling locations, including the Gulang, Lingtai and Xifeng loess sections in Central Asia, the Sanbao and Hulu stalagmites in East Asia, sediment cores V21-146 and H3571 in the North Pacific Ocean, and the NEEM ice core in Greenland.**

**
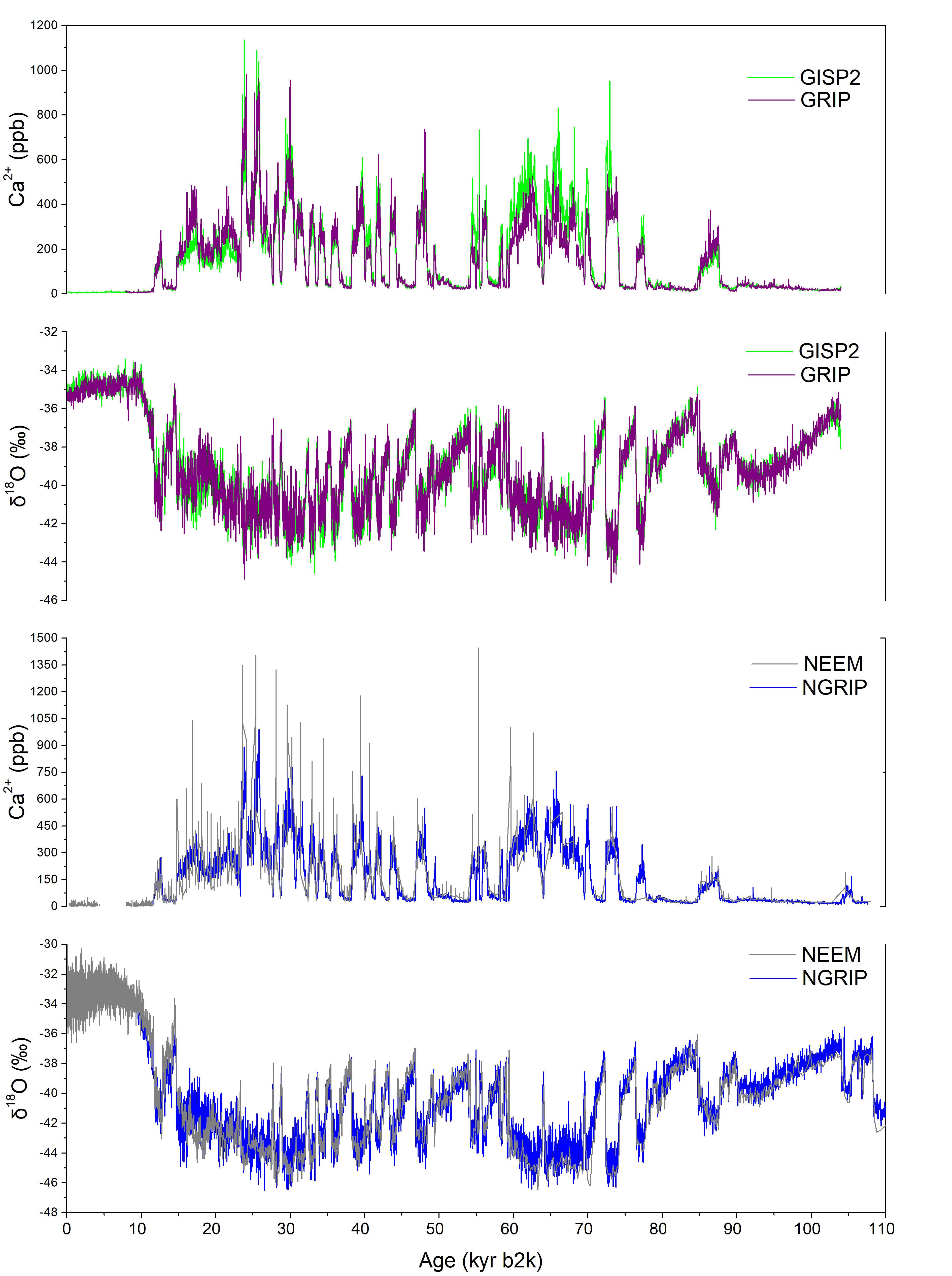
**

**Supplementary Figure 2. δ^18^O and Ca^2+^ values in the Greenland GRIP, GISP2, NGRIP and NEEM deep ice cores (δ^18^O and Ca^2+^ data from the GRIP, GISP2, NGRIP ice cores are from www.iceandclimate.dk/data).**

**
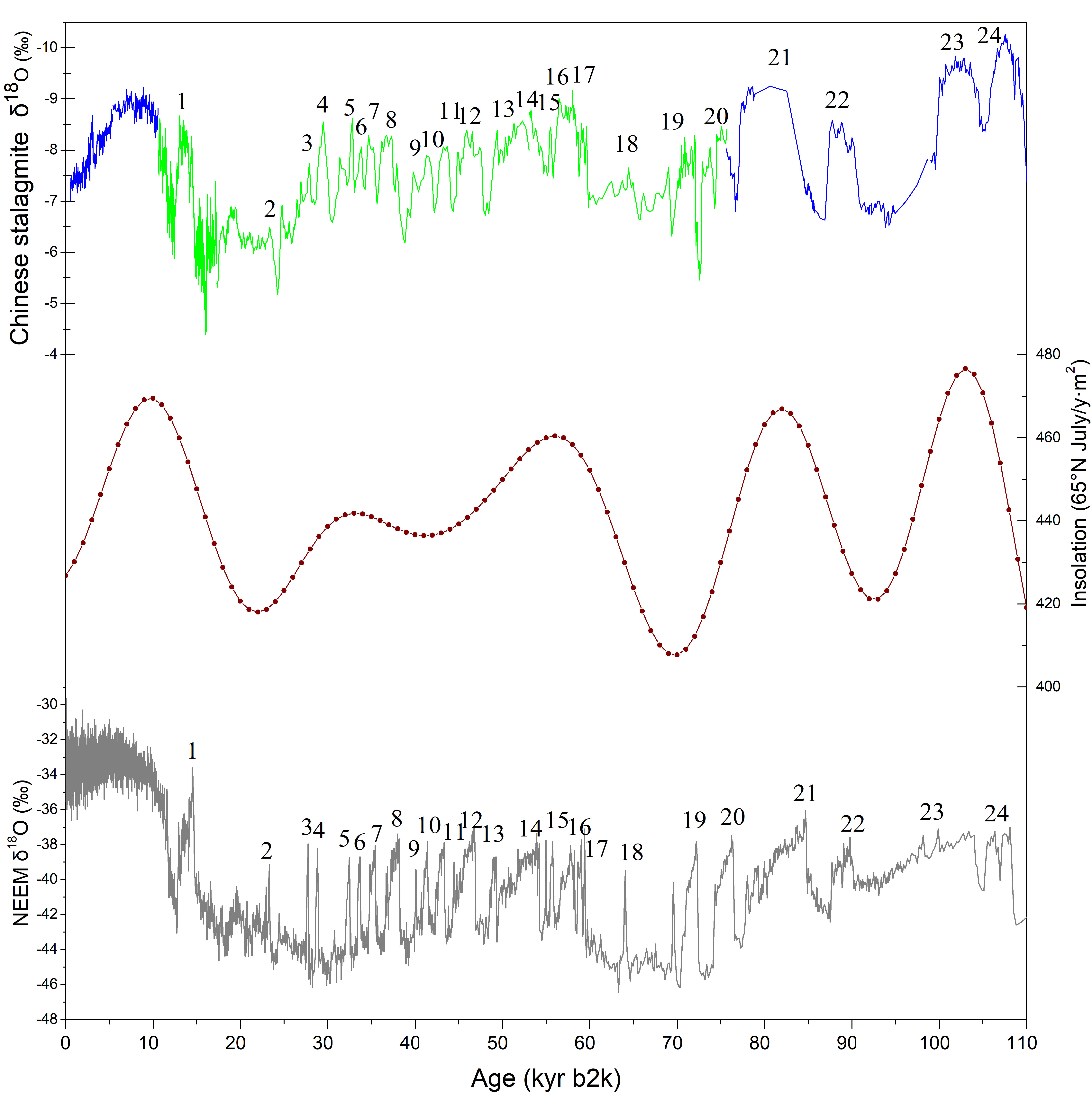
Supplementary Figure 3. δ^18^O values in the NEEM deep ice core (gray), Hulu (green)/Sanbao (blue) stalagmites [7, 8] (note that the δ^18^O values in the Sanbao stalagmite were calibrated at 1.6 ‰ because of the differences in elevations) and insolation 65°N July [9] (brown) versus time.**

**
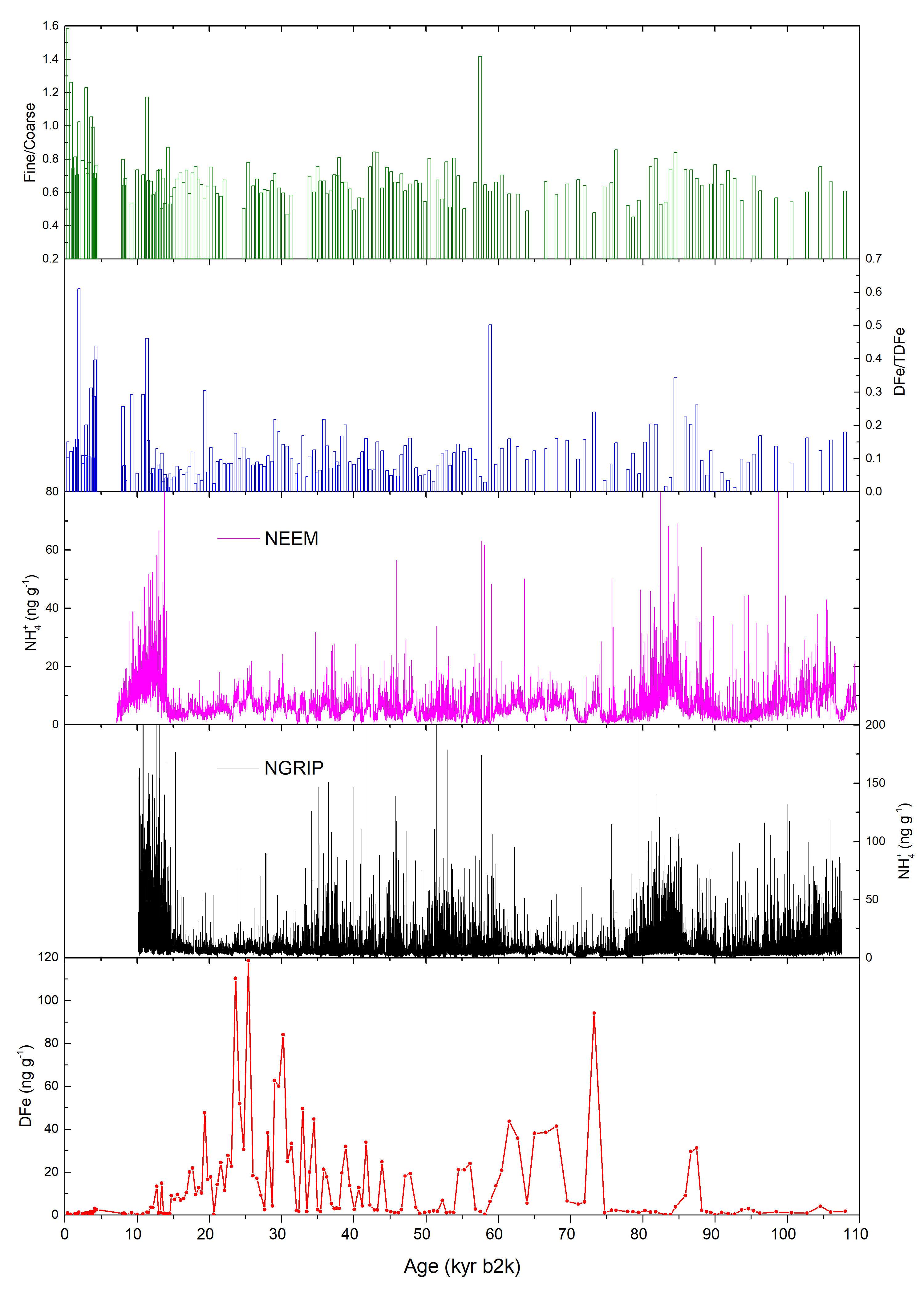
**

**Supplementary Figure 4. DFe and**
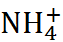
 **(black line represents NGRIP ice core from [10]) and magenta line represents NEEM ice core from [11], DFe/TDFe and Mass ratios between fine (0.8-2 μm) and coarse (2.2-5 μm) particles recorded in the NEEM ice core.**

**References**

1. Dahl-Jensen D,  Albert M R and Aldahan A *et al.* Eemian interglacial reconstructed from a Greenland folded ice core. *Nature* 2013; **493**:489–494.

2. Koffman BG, Handley MJ and Osterberg EC *et al*. Dependence of ice-core relative

trace-element concentration on acidification. *J Glaciol* 2014; **60**: 103–112.

3. Edwards R, Sedwick P and Morgan V *et al.* Iron in ice cores from Law Dome: a record of atmospheric iron deposition for maritime East Antarctica during the Holocene and Last Glacial Maximum. *Geochem Geophys Geosystems* 2006; **7**: Q12Q01.

4. Conway TM, Wolff EW and Röthlisberger R *et al*. Constraints on soluble aerosol iron flux to the Southern Ocean at the Last Glacial Maximum. *Nat Commun* 2015; **6**: 7850.

5. Gaspari V, Barbante C and Cozzi G *et al.* Atmospheric iron fluxes over the last deglaciation: climatic implications. *Geophys Res Lett* 2006; **33**: L03704.

6. Rasmussen SO, Bigler M and Blockley SP *et al.* A stratigraphic framework for abrupt climatic changes during the Last Glacial period based on three synchronized Greenland ice-core records: refining and extending the INTIMATE event stratigraphy. *Quat Sci Rev* 2014; **106**:14–28.

7. Wang YJ, Cheng H and Edwards RL *et al.* A High-Resolution Absolute-Dated Late Pleistocene Monsoon Record from Hulu Cave, China. *Science* 2001; **294**:2345–2348.

8. Wang YJ*,* Cheng H and Edwards RL *et al.* Millennial- and orbital-scale changes in the East Asian monsoon over the past 224,000 years. *Nature* 2008; **451**:1090–1093.

9. Huybers P. Early Pleistocene glacial cycles and the integrated summer insolation forcing. *Science* 2006; **313**:508–511.

10. Fischer H, Schüpbach S and Gfeller G *et al*. Millennial changes in North American wildfire and soil activity over the last glacial cycle. *Nat Geosci* 2015; **8**:723–727.

11. Schupbach S, Fischer H and Bigler M *et al.* Greenland records of aerosol source and atmospheric lifetime changes from the Eemian to the Holocene. *Nat Commun* 2018; **9**: 1476.
